# Yes-Associated Protein Regulates the Hepatic Response After Bile Duct Ligation

**DOI:** 10.1002/hep.25769

**Published:** 2012-08-08

**Authors:** Haibo Bai, Nailing Zhang, Yang Xu, Qian Chen, Mehtab Khan, James J Potter, Suresh K Nayar, Toby Cornish, Gianfranco Alpini, Steven Bronk, Duojia Pan, Robert A Anders

**Affiliations:** 1 Department of Pathology, Johns Hopkins University School of MedicineBaltimore, MD; 2 Howard Hughes Medical Institute, Johns Hopkins University School of MedicineBaltimore, MD; 3 Department of Molecular Biology and Genetics, Johns Hopkins University School of MedicineBaltimore, MD; 4 Department of Medicine, Johns Hopkins University School of MedicineBaltimore, MD; 5 Department of Medicine and Scott & White Digestive Disease Research Center, Texas A&M Health Science Center, College of Medicine, and Scott & White Hospital, and Research Service, Central Texas Veterans Health Care SystemTemple, TX; 6 Division of Gastroenterology and Hepatology, Mayo Clinic School of MedicineRochester, MN

## Abstract

Human chronic cholestatic liver diseases are characterized by cholangiocyte proliferation, hepatocyte injury, and fibrosis. Yes-associated protein (YAP), the effector of the Hippo tumor-suppressor pathway, has been shown to play a critical role in promoting cholangiocyte and hepatocyte proliferation and survival during embryonic liver development and hepatocellular carcinogenesis. Therefore, the aim of this study was to examine whether YAP participates in the regenerative response after cholestatic injury. First, we examined human liver tissue from patients with chronic cholestasis. We found more-active nuclear YAP in the bile ductular reactions of primary sclerosing cholangitis and primary biliary cirrhosis patient liver samples. Next, we used the murine bile duct ligation (BDL) model to induce cholestatic liver injury. We found significant changes in YAP activity after BDL in wild-type mice. The function of YAP in the hepatic response after BDL was further evaluated with liver-specific *Yap* conditional deletion in mice. Ablating *Yap* in the mouse liver not only compromised bile duct proliferation, but also enhanced hepatocyte necrosis and suppressed hepatocyte proliferation after BDL. Furthermore, primary hepatocytes and cholangiocytes isolated from *Yap*-deficient livers showed reduced proliferation in response to epidermal growth factor *in vitro*. Finally, we demonstrated that YAP likely mediates its biological effects through the modulation of *Survivin* expression. *Conclusion*: Our data suggest that YAP promotes cholangiocyte and hepatocyte proliferation and prevents parenchymal damage after cholestatic injury in mice and thus may mediate the response to cholestasis-induced human liver disease. (Hepatology 2012;56:1097–1107)

Human cholestatic liver diseases are characterized by cholangiocyte proliferation, hepatocellular injury, and, ultimately, liver fibrosis. Chronic cholelithiasis, primary sclerosing cholangitis (PSC), primary biliary cirrhosis (PBC), and biliary atresia are examples of cholestatic human liver diseases, as reviewed elsewhere.^1^, ^2^ Cholestasis results in bile duct injury caused by the accumulation of toxic hydrophobic bile acids, which also damages the periportal hepatocytes.^3^ In response to injury, both biliary epithelial cells (BECs) and hepatocytes will proliferate to compensate for their respective loss. Repeated injury-proliferation cycles evoke inflammation and collagen deposition, which further damage the liver and lead to fibrosis.^4^ Therefore, understanding the mechanism that controls hepatocyte and BEC proliferation and survival may suggest novel therapeutic targets to improve recovery from chronic cholestatic diseases. Although several regulatory mechanisms have been reported,^5^–^9^ the understanding of the hepatic response after biliary injury remains incomplete.

Yes-associated protein (YAP) is a transcription coactivator partnered with multiple transcription factors, including the p53 family member, p73,^10^ the Runt family member, Runx2,^11^ and the N-terminal TEA domain/transcription enhancer factor family transcription factors,^12^ among others. The activity of YAP is regulated through the phosphorylation of a conserved serine residue (S112 in mice and S127 in humans) located within the 14-3-3 binding motif, HxRxxS.^13^ The phosphoylation of YAP is controlled by the Hippo-signaling pathway, a kinase cascade that is conserved in *Drosophila* and mammals, as reviewed elsewhere.^14^ Upon phosphorylation, YAP translocates from the nucleus into the cytoplasm, where its transcriptional coactivator activity is turned off.^13^ The nuclear form of YAP is oncogenic because it can induce the expression of a class of genes that promote cell proliferation and inhibit cell death, such as the inhibitor-of-apoptosis protein (IAP) family member, *BIRC5/Survivin*,^13^ the secreted *Cysteine-rich protein connective tissue growth factor* (*CTGF*),^15^ and the epidermal growth factor (EGF) family member, *amphiregulin*.^16^ Overexpression of the YAP oncoprotein or ablation of the tumor suppressors in the Hippo pathway results in nuclear YAP accumulation, which, in turn, induces marked tissue overgrowth and frequently leads to tumorigenesis in mice.^13^, ^17^–^23^ Amplification of the *Yap* gene locus has been reported in several cancers,^24^–^31^ and overexpression of YAP has been frequently found in common solid tumors.^13^, ^32^ The correlation between YAP dysregulation and tumorgenesis has attracted intensive investigation; however, the function of YAP in non-neoplastic diseases has not been explored.

Previously, we showed that liver-specific *Yap* deficiency in the embryo affected bile duct development,^21^ which prompted us to investigate whether YAP is dysregulated in biliary disorders. In this study, we showed that YAP activity is increased in both human chronic cholestatic disorders and mice after bile duct ligation (BDL). Using the inducible *Mx1-Cre* (Cre recombinase) system, we deleted YAP in adult mice and performed BDLs. We found that *Yap* deficiency compromises BEC proliferation and blunts the regenerative response of hepatocytes. The mechanism accounting for loss of BEC proliferation was not associated with a change in Notch, Hedgehog, or Wnt signaling, but rather with loss of *Survivn* expression, whereas other hepatocyte-specific genes, such as *c-Myc* and alpha-fetoprotein (*AFP*), remained unchanged. Thus, these experiments point to a newly uncovered mechanism in controlling the hepatic response after biliary injury.

## Materials and Methods

### Human Subjects

The use of human tissue was approved by the Johns Hopkins University (Baltimore, MD) Institutional Review Board. Formalin-fixed, paraffin-embedded liver sections from patients undergoing orthotopic liver transplantation for advanced stage (3-4)^33^ biliary disease consisted of 4 patients with PSC and 7 with PBC. The 3 control healthy livers were from patients undergoing resection of liver metastasis. All material was retrieved from pathology archives in Johns Hopkins University School of Medicine.

### Animals

All animals were handled according to National Institutes of Health guidelines and protocols approved by the institutional animal care and use committee. *Yap*^*flox/flox*^ mice have been described previously^21^ and were maintained on a C57Bl/6J background. To achieve liver-specific gene deletion in the adult phase, *Yap*^*flox/flox*^ mice were injected with adenovirus expressing Cre or bred with transgenic (Tg) mice expressing Cre under the interferon-alpha-inducible *Mx1* promoter (Tg[Mx1-cre]1Cgn/J; Jackson Laboratories).^34^ All experiments were performed in male mice and paternal inheritance of *Mx1-Cre*.

### Animal Procedures

The adenoviruses, Cre-expressing adenovirus (Ade-Cre) and AdeGFP/LacZ, were prepared by David C. Johns (Johns Hopkins University). Animals were injected through the retro-orbital vein with 3 × 10^9^ plaque-forming units. Activation of the *Mx1* promoter was induced by three intraperitoneal (IP) injections of 600 μg of polyinosinic and polycytidylic acid (polyIC) (catalog no.: P1530; Sigma-Aldrich, St. Louis, MO) every other day to 5-week-old mice. One week after polyIC injection, BDL was performed as described previously.^35^, ^36^ Liver samples were harvested at indicated time points. For Fas studies, mice were injected IP with 0.165 μg/g weight of Jo-2 monoclonal antibody (catalog no.: 554255; BD Pharmingen, San Diego, CA), and the serum and liver were harvested 6 hours later.

### Primary Cell Isolation and Culture

Hepatocytes were isolated by two-step collagenase perfusion of 8- to 12-week-old mice.^37^ BECs were isolated according to the method of Vroman and LaRusso et al.^38^ Cell proliferation and culture details are presented in the Supporting Materials.

### Histology and Immunostaining

Freshly dissected liver was fixed, processed, and paraffin-embedded in the Department of Pathology Reference Histology lab according to standard protocols. Five-micron paraffin-embedded sections were stained with hematoxylin and eosin (H&E) or processed further for immunostaining. Immunohistochemical (IHC) and immunofluorescent staining were performed according to the protocols provided by the manufacturers of the respective antibodies. Antibodies that were used are listed in Supporting Table 1. The DAB+ (catalog no.: 00-2014; Invitrogen, Carlsbad, CA) visualization system was used for IHC.

**Table 1:** Antibodies Used for Immunostaining

Antibody	Source/Catalog #/Dilution
Ki67	DAKO, M7249, 1/25
CK19	DSHB, Troma III, 1/50
CK7	DAKO, M7018, 1/50
YAP (for human liver)	Epitomics, 2060-1, 1/200
YAP (for mouse liver)	Cell signaling, 4912, 1/100
TUNEL	Roche, 11684795910
Envision anti-rabbit	DAKO, P0450, 1/50
Rabbit anti-Rat	DAKO, P0450, 1/50
Alexa488 conjugated goat anti-rat	Invitrogen, A11006, 1/200
Alexa568 conjugated goat anti-rabbit	Invitrogen, A11001, 1/200

### Quantification of Parenchymal Necrosis Area and Number of BECs After BDL

H&E-stained liver sections were used to measure the areas of necrosis using ImageJ software (National Center for Biotechnology Information [NCBI], Bethesda, MD). Five 2× objective fields were randomly chosen, imaged, and the percentage of necrosis area/total area was then calculated.

Liver sections were stained with cytokeratin (CK)19 to highlight BECs. To exclude the difference between dilated and undilated bile ducts, we measured the perimeter of each bile duct to evaluate the BEC numbers. The perimeter of each bile duct was measured with ImageJ software (NCBI). Five 4× objective fields were randomly chosen, imaged, and the bile duct perimeters were calculated by adding the respective numbers of each field.

### RNA Isolation and Real-Time Polymerase Chain Reaction

Cellular RNA was extracted using the RNeasy kit (catalog no.: 74104; Qiagen, Venlo, The Netherlands), reverse-transcripted, and subjected to real-time quantitative polymerase chain reaction (PCR), as described in the Supporting Materials.

### Protein Lysate and Western Blotting Analysis

Tissues, isolated hepatocytes, or bile ducts were lysed in radioimmunoprecipitation assay buffer (150 mM of NaCl, 50 mM of Tris-HCl [pH7.4], 1% NP-40, 0.5% sodium deoxycholate, and 0.1% sodium dodecyl sulfate) with protease inhibitor (catalog no.: 10925700; Roche, Mannheim, Germany) and phosphatase inhibitor (catalog no.: 78420; Thermo Scientific, Rockford, IL). Proteins were separated on 4%-12% Bis-Tris gels (catalog no.: NP0322BOX; Invitrogen) and transferred onto polyvinylidene fluoride membranes. Blots were probed with antibodies against YAP (catalog no.: 4912; Cell Signaling Technology, Inc., Danvers, MA) and phospho-YAP (Ser127) (catalog no.: 4911; Cell Signaling Technology) and normalized by glyceraldehyde 3-phosphate dehydrogenase (catalog no.: G9545; Sigma-Aldrich). Signals were detected and quantified by the Molecular Imager Gel Doc XR System (Bio-Rad, Hercules, CA).

### Serum Alanine Aminotransferase Measurements

Serum levels of alanine aminotransferase (ALT) were measured using commercially available kits (product nos. 68-D) from Biotron Diagnostic Inc. (Hemet, CA), according to the manufacturer's protocol.

## Results

### Bile Ductular Reactions in Human PSC and PBC Diseased Livers Show Increased Nuclear YAP Expression and Activity

**Fig. 1:**
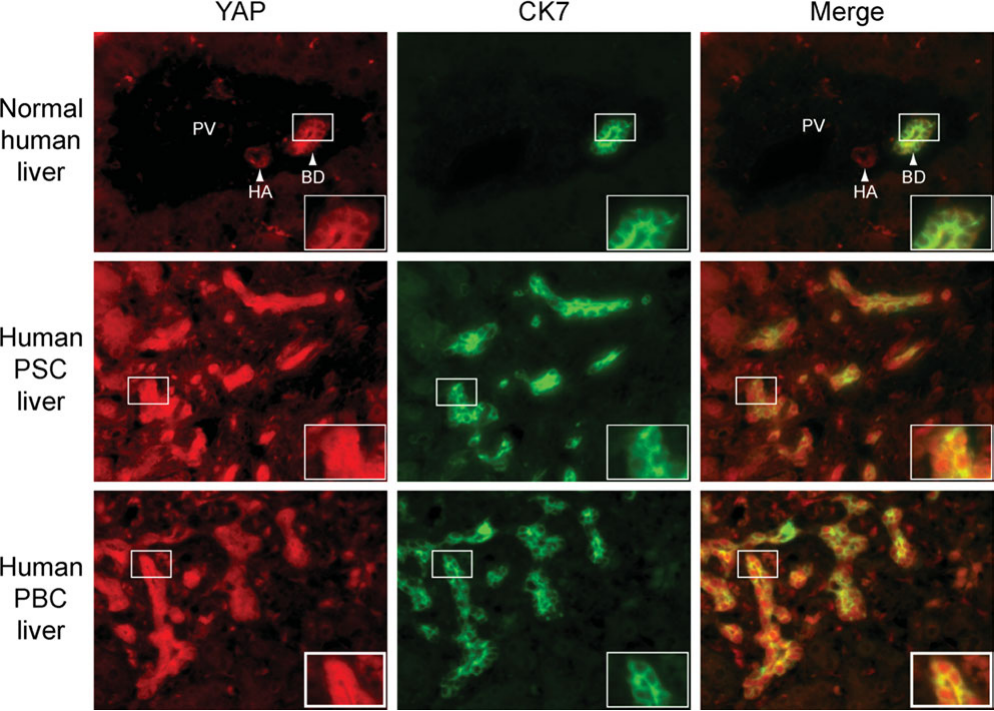
Bile ductular reactions in human PSC and PBC diseased livers show increased nuclear YAP expression and activity. Healthy human liver as well as PSC and PBC patient liver sections were costained with YAP and BEC membranous marker CK7. Upper panels, healthy human liver, showing YAP on the plasma membrane of the portal-tract–associated bile duct (BD) epithelial cells. Middle panel, PSC patient liver. Bottom panel, PBC patient liver. Note uniformly elevated YAP staining throughout the cell, including the nucleus, in BECs of bile ductular reactions. Insets are higher magnifications of representative areas. PV, portal vein, HA, hepatic artery. Original magnification (×20 objective), insets (×40 objective).

Liver sections from control patients without chronic cholestatic liver disease and patients with advanced stage (3-4) PSC or PBC were examined for YAP protein expression. In the healthy human liver, YAP staining was localized to the portal tract bile duct epithelium and the endothelium of the hepatic artery (Fig. 1, top panel). There was no specific staining of YAP in the hepatocytes. We looked in more detail at the interlobular bile ducts and found strong YAP staining on the plasma membrane of BECs, but little YAP staining in the nucleus, as revealed by colocalization with BEC membranous marker CK7. However, in diseased cholestatic PSC and PBC livers, we found the uniform strong expression of YAP in the periportal bile ductular reactions associated with each of the patient samples (N = 11). In contrast to the predominant membrane localization of YAP in BECs of the healthy human liver, the BECs in the ductular reactions of the cholestatic livers showed uniformly YAP staining throughout the cell, including the nucleus (Fig. 1, middle and bottom panels). Furthermore, YAP is expressed in small individual cells in the periportal region. It is possible that these represent liver-progenitor–like cells. Given the role of nuclear YAP in promoting active transcription,^13^ these findings suggest that YAP activity in BECs may participate in the bile ductular reactions observed in human cholestatic liver diseases.

### Induction of YAP in the Murine Liver After Experimentally Induced Cholestasis

**Fig. 2:**
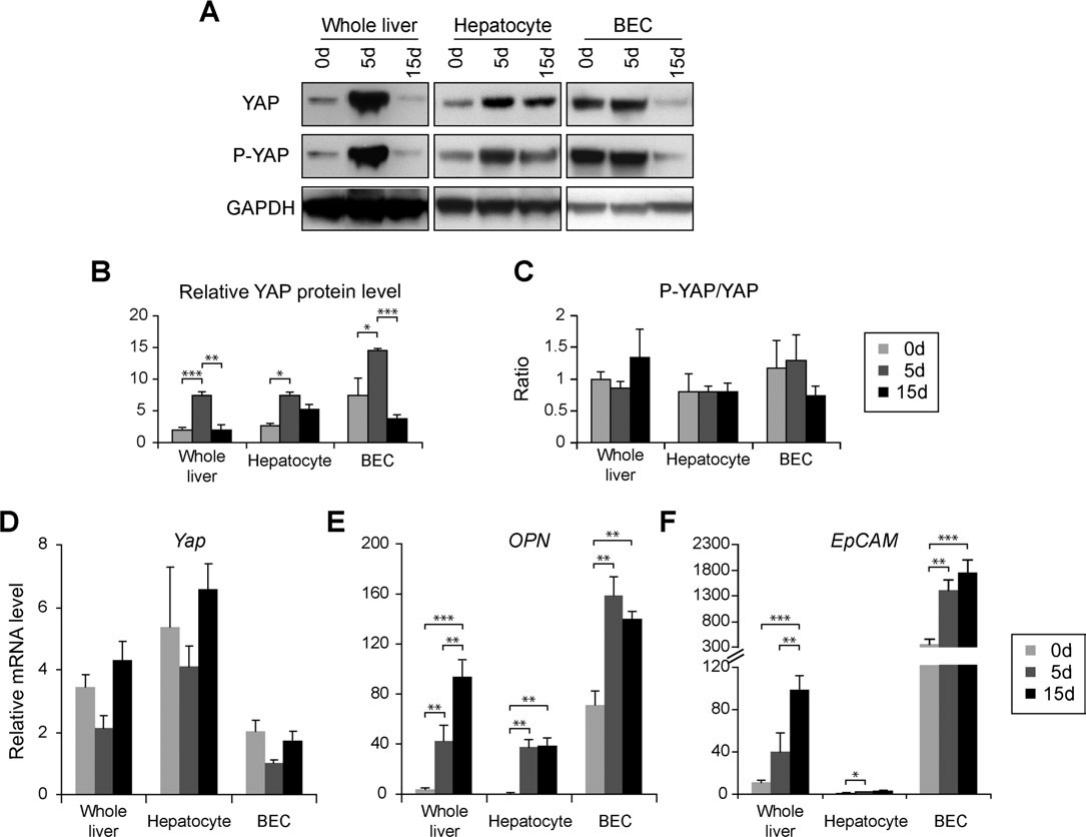
Induction of YAP in the murine liver after experimentally induced cholestasis (A) western blotting analysis. Protein extracts from whole liver, hepatocyte, and BECs of WT mice immediately (0 d) and 5 days (5 d) and 15 days (15 d) after BDL were probed with the indicated antibodies. Note the increase of YAP and phosphorylated YAP (P-YAP) levels in livers 5 days post-BDL. (B and C) YAP protein levels and P-YAP/YAP ratio are quantified in the indicated graphs. Values represent means ± standard error of the mean (SEM) (n = 3-4). *P < 0.05; **P < 0.01; ***P < 0.001; *t* test. (D-F) Real-time PCR analysis. mRNAs from whole liver, hepatocyte, and BECs of WT mice immediately (0 d), 5 days (5 d), and 15 days (15 d) after BDL were probed with the indicated genes. Note the unchanged *Yap* mRNA levels in all three components (D) and steadily increasing mRNA levels of BEC markers *OPN* (E) and *EpCAM* (F) after BDL in the whole liver. Values represent means ± SEM (n = 3-4). *P < 0.05; **P < 0.01; ***P < 0.001; *t* test.

To study the potential role of YAP in a cholestatic liver, we adopted the well-established BDL animal model of human cholestatic liver disease.^39^ We performed BDL on mice and harvested the liver tissue immediately after ligation (day 0), limiting any physiological change within the liver. We also harvested livers at 5 days post-BDL, which corresponds to the peak of BEC and hepatocyte proliferation, as well as 15 days post-BDL, when the ductular reactions have ceased.^40^ We examined the hepatic YAP expression in the liver after BDL. Western blotting analysis detected an increase of YAP protein levels in the whole liver, hepatocytes, and BECs 5 days after BDL (Fig. 2A,B). This increase of YAP protein was not the result of increased transcription, because *Yap* messenger RNA (mRNA) was unchanged (Fig. 2D). In contrast, mRNA levels of BEC-enriched marker osteopontin (*OPN*) and epithelial cell adhesion molecule (*EpCAM*)^21^ increased steadily in the whole liver from days 0 to 15 post-BDL (Fig. 2E,F). Because BECs showed significantly higher *OPN* and *EpCAM* mRNA levels than hepatocytes at all time points (Fig. 2E,F), the steady increase in *OPN* and *EpCAM* mRNA levels in the whole liver likely reflects an increase in the number of BECs. Therefore, *OPN* and *EpCAM* mRNA levels from whole liver can serve as an indirect measure of BEC mass. Even though there was an overall increase in YAP activity, reflected in the significant increase in total YAP protein 5 days post-BDL, the Hippo pathway's kinase cascade was unperturbed, because the ratio of phosphorylated YAP to total YAP protein remained unchanged (Fig. 2C). Consistent with this finding, immunostaining showed that YAP protein levels were increased in both the nucleus and cytoplasm of hepatocytes and BECs 5 days post-BDL, compared to those at days 0 and 15 post-BDL (Supporting Fig. 1).

### Yap *Deficiency Compromises Bile Ductular Reaction After BDL*

**Fig. 3:**
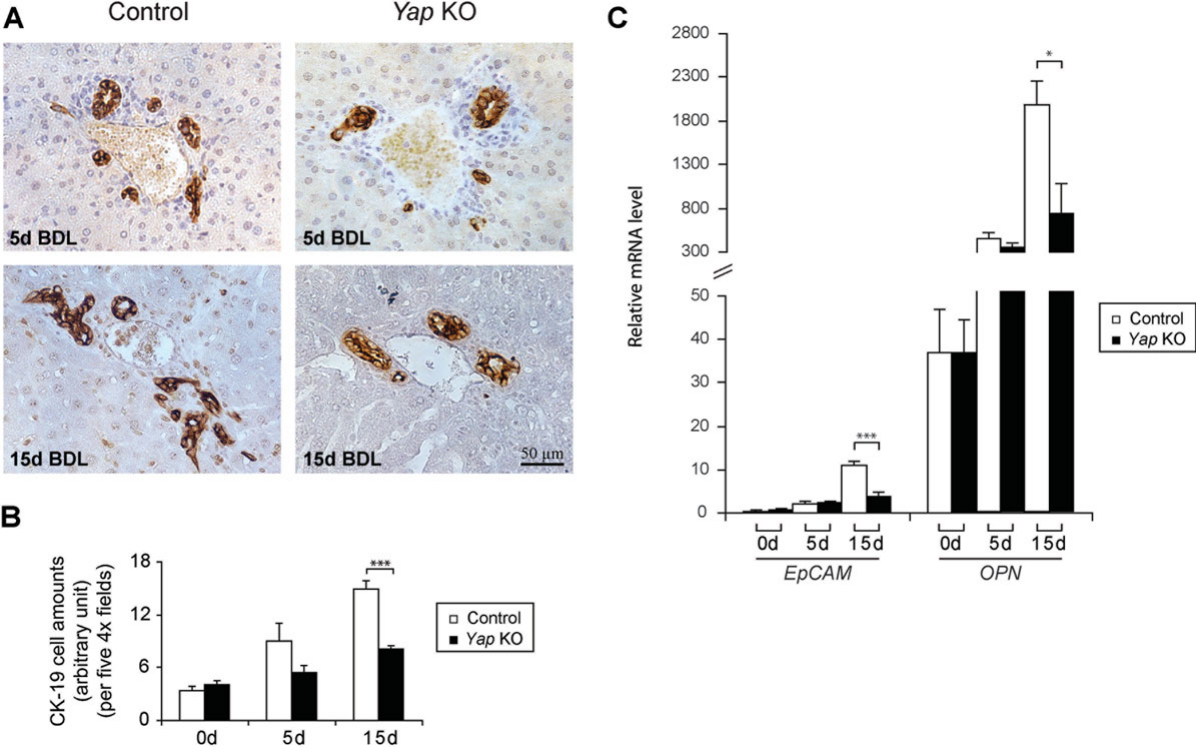
*Yap* deficiency compromises bile ductular reaction after BDL. (A) Representative liver section stained for the BEC marker, CK19, after BDL. (B) Quantification of CK19-positive cells. Values represent means ± standard error of the mean (SEM) (n = 3-5). ***P < 0.001; *t* test. (C) Real-time PCR analysis. mRNAs from control and *Yap*-deficient livers immediately (0 d), 5 days (5 d), and 15 days (15 d) after BDL were probed with BEC markers *EpCAM* and *OPN*. Note the decreased mRNA levels of *EpCAM* and *OPN* in *Yap*-deficient livers, relative to control livers, 15 days post-BDL. Values represent means ± SEM (n = 3-5). *P < 0.05; ***P < 0.001; *t* test.

To test whether this increased YAP activity is critical for the hepatic response after BDL, we performed BDL in mice with liver-specific deletion of *Yap* in adult mice. The previously reported *Alb*-*Cre;Yap*^*flox/flox*^ is not suitable for these studies, because these mice develop an abnormal embryonic biliary system.^21^ We achieved deletion of *Yap* in the adult liver by injecting *Ade-Cre* into *Yap*^*flox/flox*^ mice or injecting polyIC into *Mx1-Cre;Yap*^*flox/flox*^ mice. Both methods resulted in the efficient suppression of YAP expression in the adult liver, with no observable phenotypic changes in the liver (Supporting Figs. 2 and 3). These data suggest that the deletion of YAP in the adult mouse does not induce baseline changes in BECs or hepatocytes. Because *Mx1-Cre;Yap*^*flox/flox*^ mice show slightly better suppression of *Yap* mRNA and avoid the potential complication of adenovirus-induced hepatitis,^41^ all subsequent experiments detailed polyIC-treated *Mx1-Cre;Yap*^*flox/flox*^ with *Yap*^*flox/flox*^ littermate controls. After BDL, it has been shown that there is proliferation of BECs at the periportal interface between the biliary tree and hepatocytes,^40^, ^42^ which can be highlighted using CK19 IHC staining. We found that the number of BECs increased in both *Yap*^*flox/flox*^ and *Mx1-Cre;Yap*^*flox/flox*^ livers 5 days post-BDL and were comparable in both genotypes (Fig. 3A,B and Supporting Fig. 4 5d). However, at 15 days post-BDL, there were significantly fewer BECs in *Mx1-Cre;Yap*^*flox/flox*^ livers, compared to the control *Yap*^*flox/flox*^ livers (Fig. 3A,B and Supporting Fig. 4 15d). In agreement with the histological and IHC analysis, there was a progressive increase in mRNA levels of BEC markers *EpCAM* and *OPN* from days 5 to 15 post-BDL in control *Yap*^*flox/flox*^, but not in *Mx1-Cre;Yap*^*flox/flox*^ livers (Fig. 3C). Thus, loss of YAP activity compromises the bile ductular reaction after BDL.

### YAP Is Required for BEC Proliferation After BDL

**Fig. 4:**
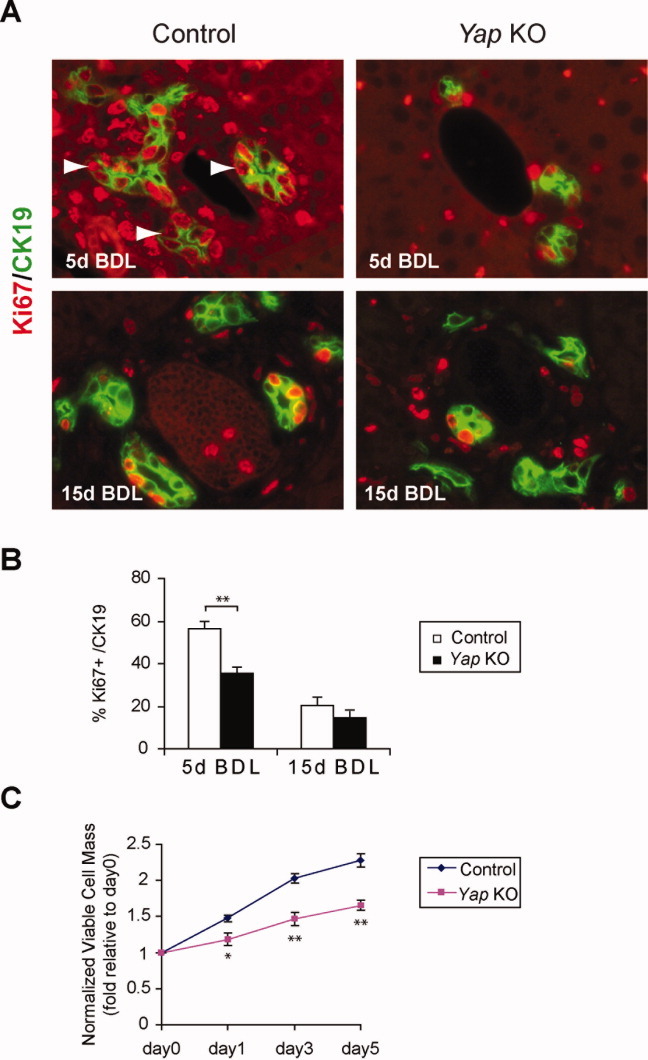
YAP is required for BEC proliferation after BDL. (A) Costaining of proliferation marker Ki67 (red, nucleus) and BEC marker CK19 (green, membrane). White arrowheads point to representative proliferating BECs, which express both CK19 and Ki67. Note the reduced percentage of proliferating BECs in *Yap*-deficient livers, compared to control livers, 5 days post-BDL. (B) Quantification of the percentage of dual positive Ki67^+^/CK19^+^ BECs to total BECs (CK19^+^ only). Values represent means ± standard error of the mean (SEM) (n = 3-5). **P < 0.01; *t* test. (C) Reduced proliferation response of *Yap*-deficient BECs to EGF stimulation. BECs isolated from control and *Yap*-deficient livers were cultured *in vitro* in the presence of EGF. Viable cell numbers were measured at indicated time points and plotted as fold of viable cells relative to day 0. Values are means ± SEM (n = 3). **P < 0.01; ***P < 0.001; *t* test.

One of the possible sources for the bile ductular reactions is the proliferation of preexisting BECs.^43^ It has been demonstrated that BEC proliferation peaks during the first 5 days post-BDL and remains at a low level thereafter.^40^ We therefore sought to measure whether there was reduced BEC proliferation in the *Mx1-Cre;Yap*^*flox/flox*^ liver after BDL. We found that the number of Ki67-positive BECs was significantly reduced in *Mx1-Cre;Yap*^*flox/flox*^ livers at day 5 post-BDL, compared to the control *Yap*^*flox/flox*^ livers (Fig. 4A,B). In contrast, no apoptotic BECs were seen in both *Yap*^*flox/flox*^ and *Mx1-Cre;Yap*^*flox/flox*^ livers at either day 5 or 15 post-BDL (Supporting Fig. 5). To confirm that the compromised BEC proliferation post-BDL is a direct result of *Yap* deficiency, we isolated and cultured primary BECs from control and *Mx1-Cre;Yap*^*flox/flox*^ livers and compared their proliferation response to EGF stimulation. Compared with wild-type (WT) BECs, *Yap*-deficient BECs showed a significantly decreased proliferation rate (Fig. 4C). These findings suggest that *Yap* deficiency compromises cholestasis-induced bile ductular reaction as a result of impaired BEC proliferation, rather than enhanced apoptosis.

### Yap-*Deficient Hepatocytes Are More Susceptible to Injury*

**Fig. 5:**
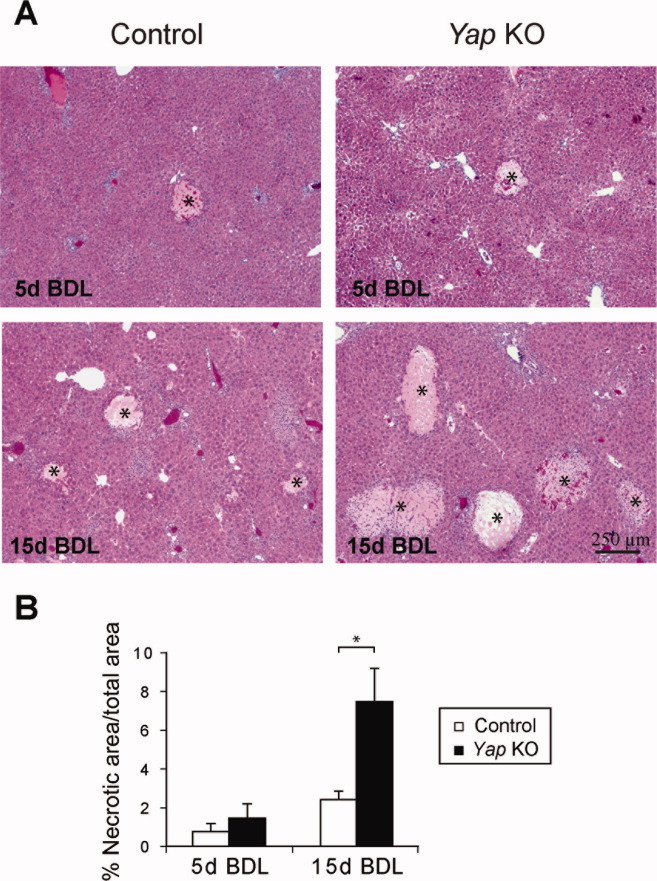
*Yap*-deficient hepatocytes are more susceptible to injury. (A) Representative H&E staining of control and *Yap*-deficient livers 5 and 15 days post-BDL. Regions of hepatocellular necrosis are indicated by asterisks. (B) Quantification of infracted area as a percentage of total area. Values represent means ± standard error of the mean (n = 3-5). *P < 0.05; *t* test.

After BDL, the *Mx1-Cre;YAP*^*flox/flox*^ mice developed ascites and 35% died within 15 days, whereas all control littermates showed no ascities or mortality. Liver histology revealed significantly more hepatocellular necrosis in *Mx1-Cre;Yap*^*flox/flox*^ than in *Yap*^*flox/flox*^ livers at day 15 post-BDL, with essentially no difference between the two groups 5 days post-BDL (Fig. 5A,B). To further test whether *Yap* deficiency compromised hepatocyte survival, we challenged the *Mx1-Cre;Yap*^*flox/flox*^ and *Yap*^*flox/flox*^ mice with Jo-2 antibody, a Fas agonist and a potent hepatocellular apoptotic stimulus.^44^, ^45^ Six hours after Jo-2 antibody injection, we observed significantly more apoptotic and terminal deoxynucleotidyl transferase dUTP nick end labeling–positive hepatocytes in *Mx1-Cre;Yap*^*flox/flox*^ livers, compared to control livers (Supporting Fig. 6A,B). Consistently, we observed a significantly higher serum ALT level in *Mx-1Cre;Yap*^*flox/flox*^ mice, compared to control *Yap*^*flox/flox*^ mice (Supporting Fig. 6C). Taken together, these data support that *Yap* deficiency compromises hepatocyte survival, which is in agreement with our previous observation that overexpression of YAP in hepatocytes confers resistance to Jo-2-induced hepatocyte apoptosis.^13^, ^17^

### Loss of YAP Delays Hepatocyte Proliferation After BDL

**Fig. 6:**
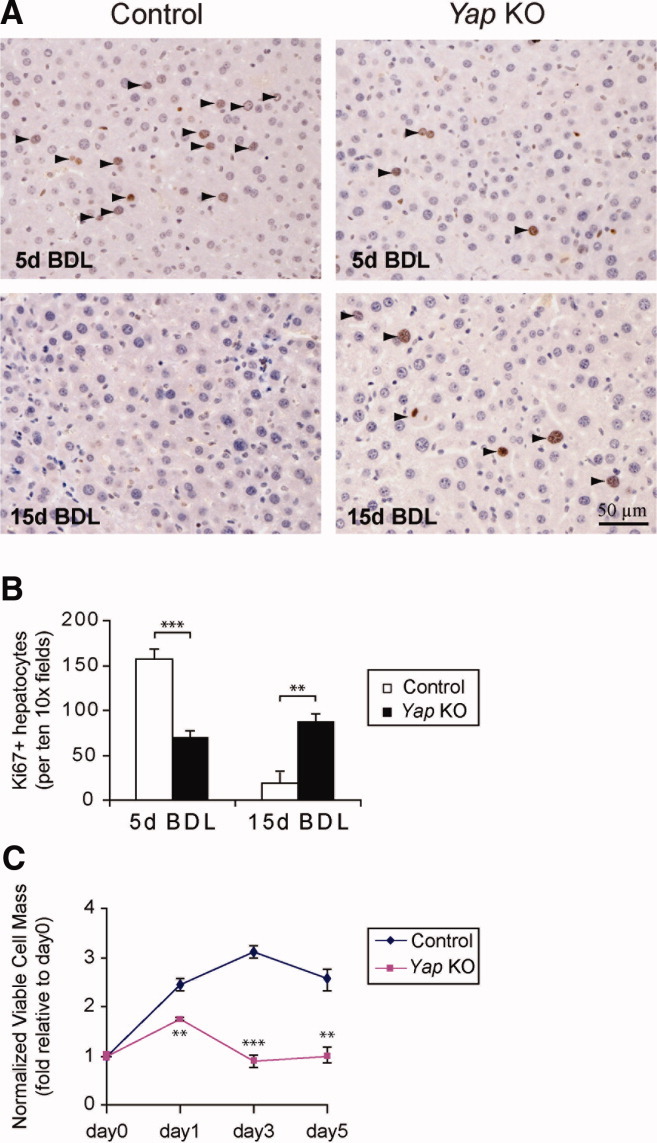
Loss of YAP delays hepatocyte proliferation after BDL. (A) Ki67 staining of control and *Yap*-deficient livers 5 and 15 days post-BDL. Black arrowheads point to Ki67^+^ hepatocyte nuclei. (B) Quantification of Ki67^+^ hepatocytes. Values represent means ± standard error of the mean (SEM) (n = 3-5). **P < 0.01; ***P < 0.001; *t* test. (C) Reduced hepatocyte proliferative response to EGF stimulation. Hepatocytes isolated from control and *Yap*-deficient livers were cultured *in vitro* in the presence of EGF. Viable cell numbers were measured at indicated time points and plotted as fold of viable cells relative to day 0. Values are means ± SEM (n = 3). *P < 0.05; **P < 0.01; *t* test.

At day 5 post-BDL, which normally corresponds to peak of hepatocyte proliferation,^40^*Mx1-Cre;Yap*^*flox/flox*^ livers showed a significant reduction in proliferating hepatocytes, compared to control *Yap*^*flox/flox*^ livers (Fig. 6A,B 5d). This difference was unlikely the result of the degree of injury, because at day 5 post-BDL, hepatocyte necrosis was similar in *Yap*^*flox/flox*^ and *Mx1-Cre;Yap*^*flox/flox*^ livers (Fig. 5A,B). On the other hand, at 15 days post-BDL, hepatocyte proliferation was apparent in *Mx1-Cre;Yap*^*flox/flox*^ livers, but not in control *Yap*^*flox/flox*^ livers (Fig. 6A,B 15d). This likely represents a kinetic delay in regenerative proliferation of hepatocytes, because there is no difference in the final liver-to-body-weight ratio in *Mx1Cre;Yap*^*flox/flox*^ (8.81% ± 0.51%) and *Yap*^*flox/flox*^ (8.46% ± 0.86%). To study whether YAP is directly involved in modulating hepatocyte proliferation, we isolated primary hepatocyte from *Yap*^*flox/flox*^ and *Mx1-Cre;Yap*^*flox/flox*^ livers and cultured them *in vitro* in the presence of EGF. The proliferation response of primary hepatocytes to EGF was significantly attenuated as a result of *Yap* deficiency (Fig. 6C), which suggested that YAP plays a direct role in delaying hepatocyte proliferation after BDL.

### YAP Mediates Survivin mRNA Expression After BDL

**Fig. 7:**
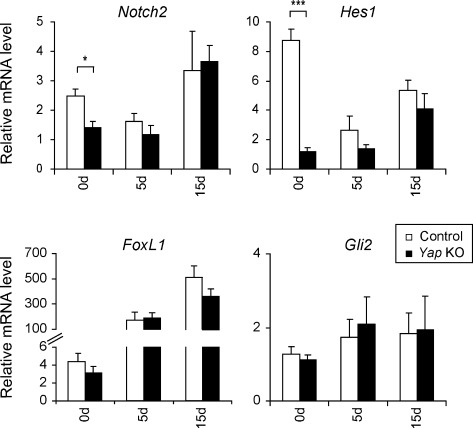
mRNA expression of critical BEC mediators in *Yap*-deficient mice after BDL. Real-time PCR analysis for mRNA levels of critical mediators of BEC development and proliferation from control and *Yap*-deficient livers immediately (0 d) or 5 days (5 d) and 15 days (15 d) after BDL. Values represent means ± standard error of the mean (n = 3-5). *P < 0.05; ***P < 0.001; *t* test.

To begin to understand how YAP might control bile duct proliferation, we examined pathways that are known to play critical roles in BEC development. We found no significant changes in the mRNA levels of the *Notch2* receptor or its target gene, *Hes1*, at 5 or 15 days after BDL. However, both genes showed a slightly lower basal line expression in *Mx1-Cre;Yap*^*flox/flox*^, compared to control *Yap*^*flox/flox*^, mice (Fig. 7).^6^, ^46^ Similarly, there was no difference between *Yap*^*flox/flox*^ control and *Mx1-Cre;Yap*^*flox/flox*^ at any of the time points after BDL for the Hedgehog target genes, *Gli2* or *FoxL1*, both of which have been reported to be critical in cholestasis-mediated bile duct proliferation (Fig. 7).^7^, ^9^

**Fig. 8:**
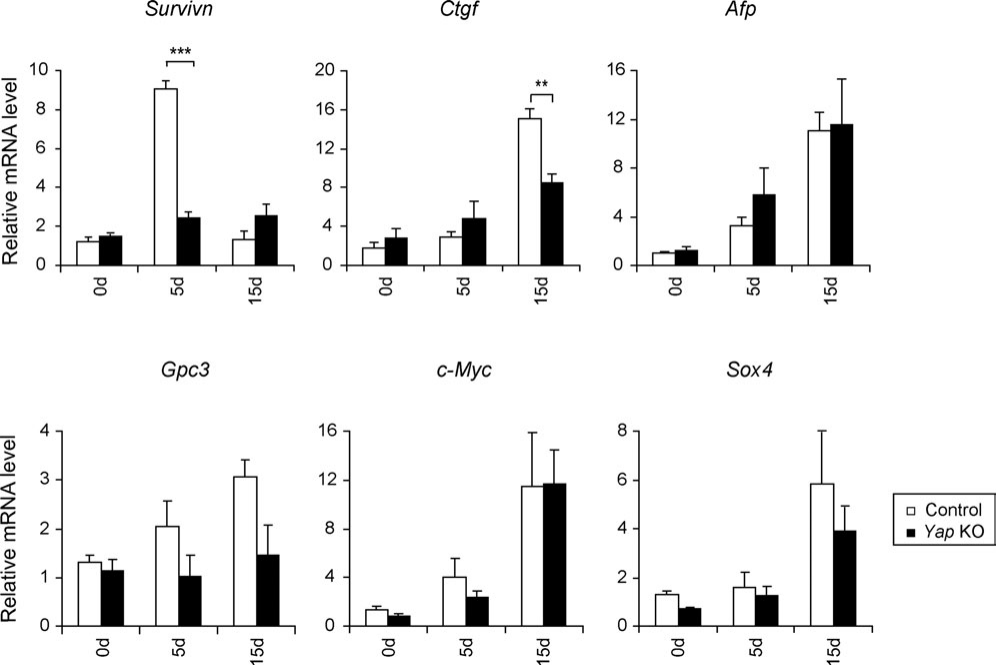
YAP mediates *Survivin* mRNA expression after BDL. Real-time PCR analysis for mRNA levels genes, which are transcriptionally up-regulated in YAP Tg livers from control and *Yap*-deficient livers immediately (0 d), 5 days (5 d), or 15 days (15 d) after BDL. Note *Survivin* mRNA levels peak at day 5 post-BDL in WT livers, and this peak was suppressed in *Yap*-deficient livers. Values represent means ± standard error of the mean (n = 3-5). **P < 0.01; ***P < 0.001; *t* test.

Next, we evaluated a set of genes' mRNA that have previously been reported to be up-regulated in YAP Tg livers, including *Survivin, Ctgf, Afp, Gpc3, c-Myc, Sox4, Opn*, and *EpCam*.^13^ We determined the mRNA expression of these genes in *Yap*^*flox/flox*^ and *Mx1-Cre;Yap*^*flox/flox*^ livers at days 0, 5, and 15 post-BDL and correlated the findings with YAP protein levels (Figs. 3C and 8). We surmised that the ideal YAP target gene(s) should have a peak expression that matches the induction of YAP protein levels in WT livers at day 5 post-BDL which should also be significantly suppressed in *Yap*-deficient livers. Among those candidate genes, only *Survivin* met the above-mentioned criteria. All other candidates showed peak gene expression at day 15 post-BDL, which more likely reflects progress of the bile ductular reaction (Fig. 3C for *EpCam* and *Opn*, and Fig. 8 for all others). Given *Survivin*'s well-documented roles in enhancing proliferation and inhibiting apoptosis,^47^, ^48^ our findings suggest that *Survivin* may be a critical mediator in YAP-mediated regenerative response after cholestatic injury.

## Discussion

In this study, we provide a promising mechanism for controlling the hepatic response to biliary obstruction. We showed that YAP, the transcription coactivator of the Hippo-signaling pathway, is activated in the livers of both human patients with biliary diseases and mice with biliary obstruction. As a transcription coactivator, YAP can activate other transcription factors to induce proliferation and antiapoptosis-associated genes. Therefore, the increased YAP activity could be critical for promoting BEC proliferation, which is an important component of the repair process that occurs after damage to bile ducts during the courses of cholestatic liver diseases. The above-described hypothesis was tested using BDL in *Yap*-deficient livers. By investigating the repair response of *Yap*-deficient livers after BDL, we showed that YAP activity is important for BEC and hepatocyte proliferation and survival of hepatocyte after biliary obstruction. Our studies identify a novel therapeutic target to enhance BEC proliferation in chronic cholestatic diseases, which could ultimately be used to prevent chronic biliary diseases from progressing to bile ductopenia.

The increase of YAP protein levels could be a universal response to tissue injury. The recently published study by Cai et al.^49^ also showed that YAP protein levels were dramatically increased in regenerating colonic crypts of murine intestine after dextran sodium sulfate (DSS)-induced injury, and that YAP activity is required for colonic regeneration after DSS treatment. Interestingly, both our study and the study of Cai et al. show that the increase in YAP protein levels in two different tissue injury models is the result of a post-transcriptional mechanism, because neither study detected an increase of *Yap* mRNA levels in regenerating tissues. Future studies should reveal the molecular underpinnings of this post-transcriptional regulation of YAP protein levels and whether it represents a more widespread mechanism in other regenerative processes.

Given its potent oncogenic function, YAP activity must be carefully regulated during and after the regenerative response to injury.^13^, ^17^, ^25^ Consistent with this hypothesis, Cai et al.^49^ and Benhamouche et al.^50^ have demonstrated a synergistic effect of tissue regeneration and defective Hippo pathway signaling in accelerating malignant transformation in murine colon and liver, respectively. Notably, the risk for developing meningioma, a tumor that frequently harbors *Nf2* mutations, is significantly increased in patients with a history of head trauma,^51^ suggesting that such synergy may also be relevant to human tumorigenesis.

Among the YAP-inducible genes in the liver, we found that only *Survivin* mRNA level correlates with YAP protein increase in WT livers after BDL and is reduced in *Yap*-deficient livers. *Survivin*, a member of the IAPs family, has been shown to suppress apoptosis through interacting with caspases^52^ and to promote cell division through interference with cell-cycle–related kinases and microtubule networks.^53^ This gene is overexpressed in many human malignancies, but is absent or present at very low levels in most non-neoplastic adult tissues.^54^ Therefore, *Survivin* appears to be a very reasonable target whose expression is induced by YAP during the regenerative response after biliary obstruction. It will be informative to investigate whether *Survivin* is required for the regeneration response after BDL with *Survivin* conditional knockout mice.^55^

## Abbreviations

Ade-Cre: Cre-expressing adenovirus
AFP: alpha-fetoprotein
ALT: alanine aminotransferase
BEC: biliary epithelial cell
BDL: bile duct ligation
CK: cytokeratin
Cre: Cre recombinase
CTGF: connective tissue growth factor
DSS: dextran sodium sulfate
EGF: epidermal growth factor
EpCAM: epithelial cell adhesion molecule
H&E: hematoxylin and eosin
IAP: inhibitor-of-apoptosis protein
IHC: immunohistochemistry
IP: intraperitoneal
mRNA: messenger RNA
NCBI: National Center for Biotechnology Information
OPN: osteopontin
PBC: primary biliary cirrhosis
PCR: polymerase chain reaction
polyIC: polyinosinic and polycytidylic acid
PSC: primary sclerosing cholangitis
Tg: transgenic
WT: wild type
YAP: Yes-associated protein
