# Supplementary figures and images for: Yes-Associated Protein Regulates the Hepatic Response After Bile Duct Ligation
Source: Hepatology. 2012 Aug 8;56(3):1097–107. doi: 10.1002/hep.25769 (PMC3431197; doi:10.1002/hep.25769)

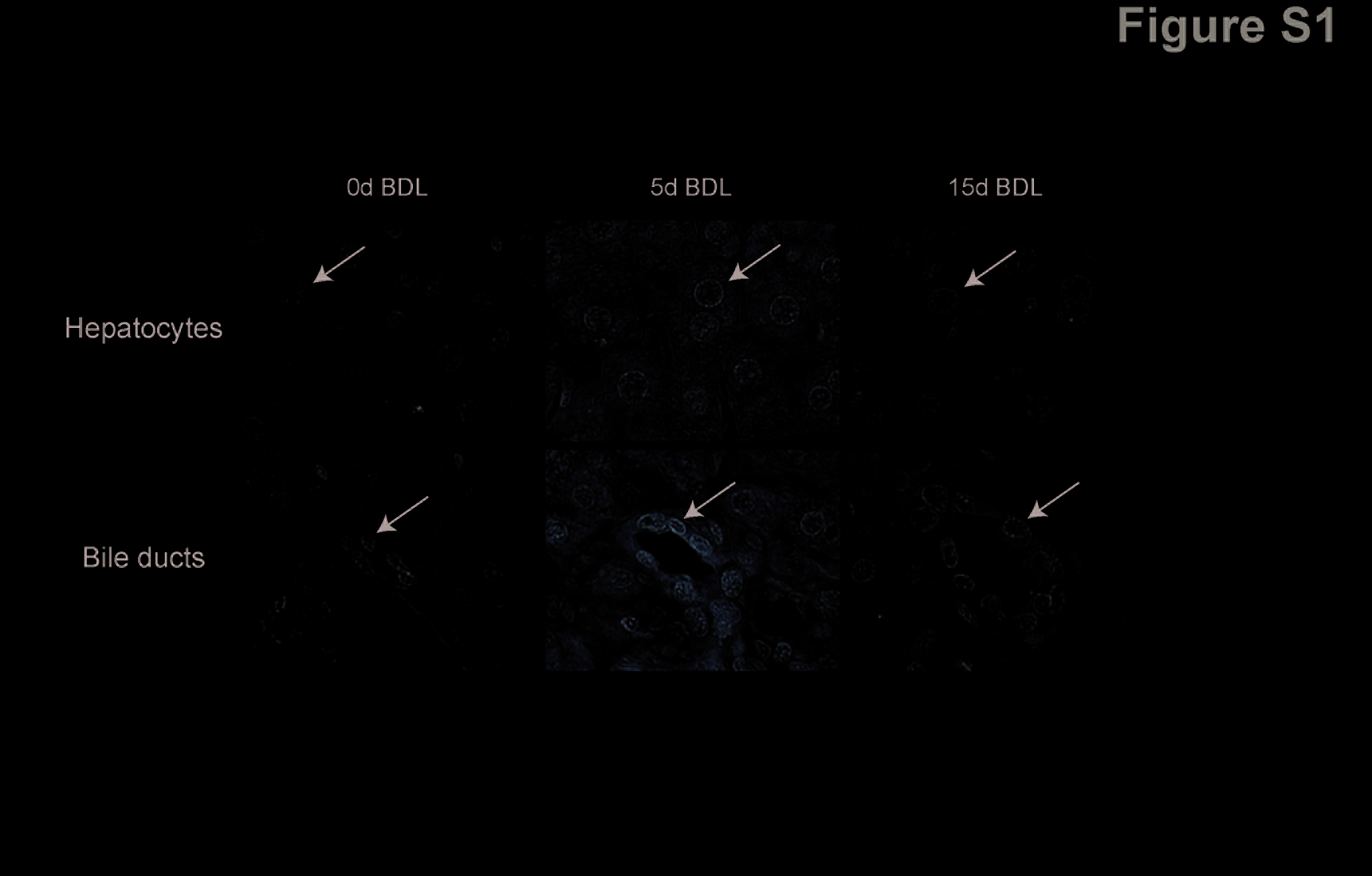

Supplement: Supplementary file 1 [file hep0056-1097-SD1.tif]

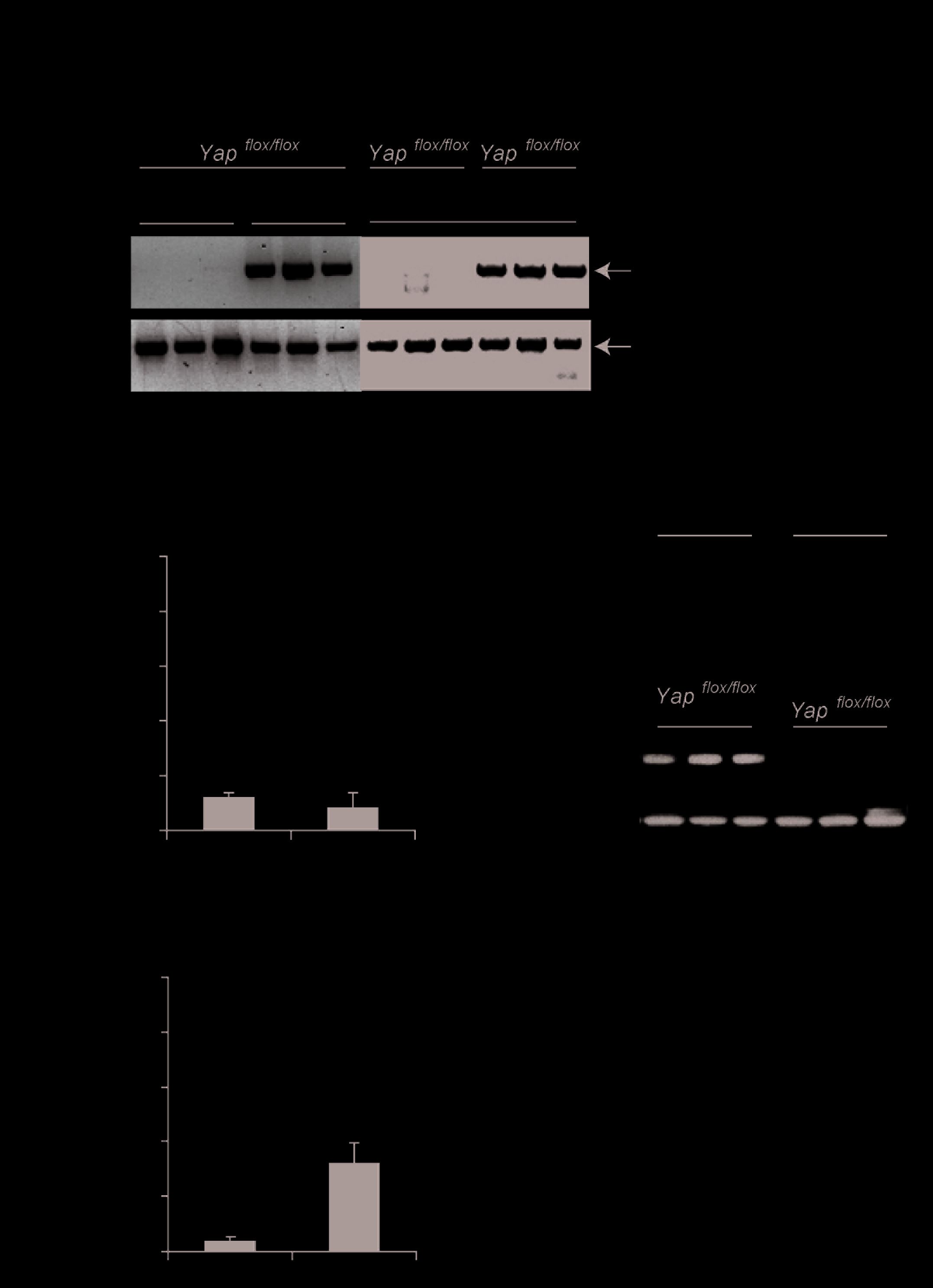

Supplement: Supplementary file 2 [file hep0056-1097-SD2.tif]

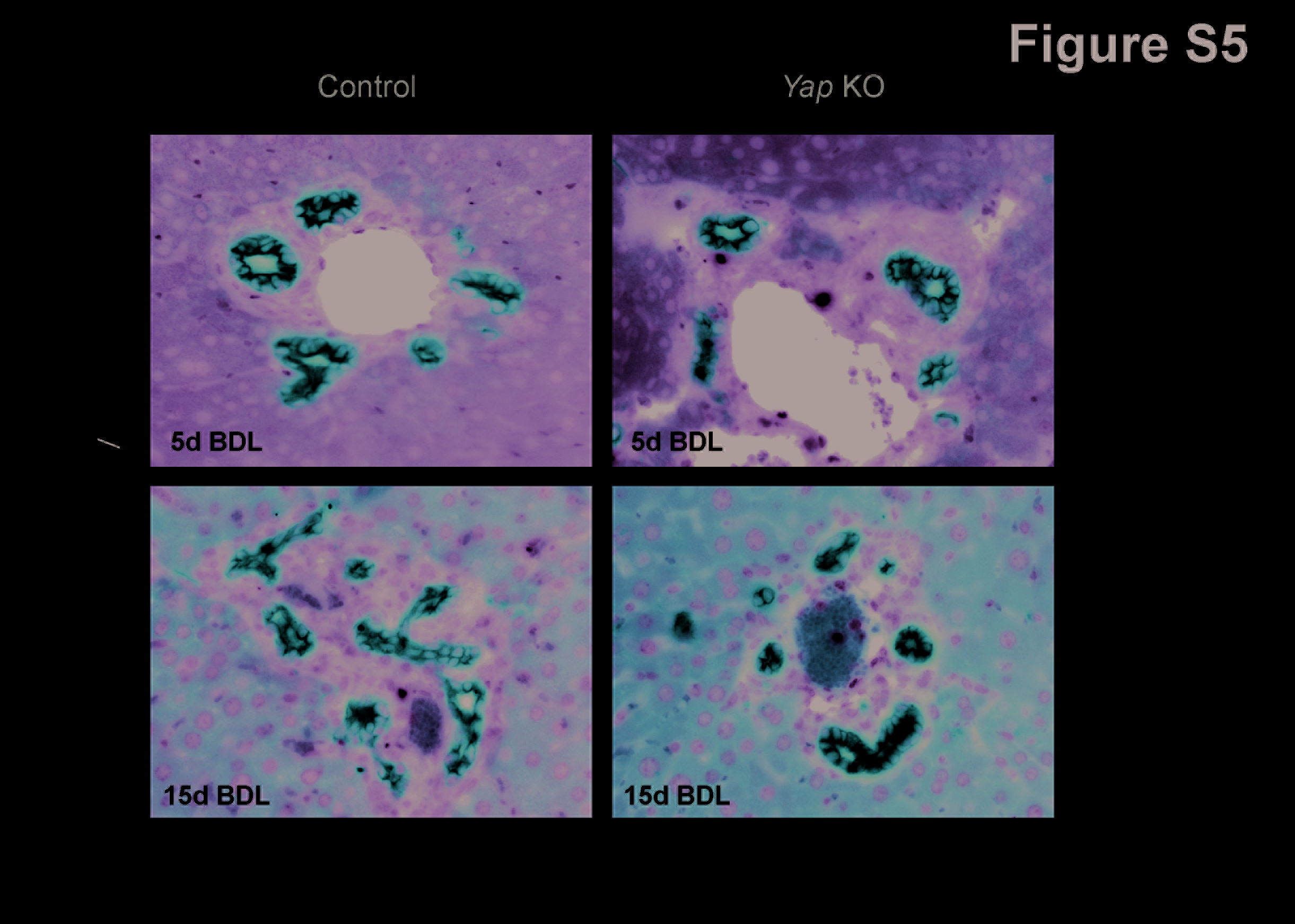

Supplement: Supplementary file 5 [file hep0056-1097-SD5.tif]

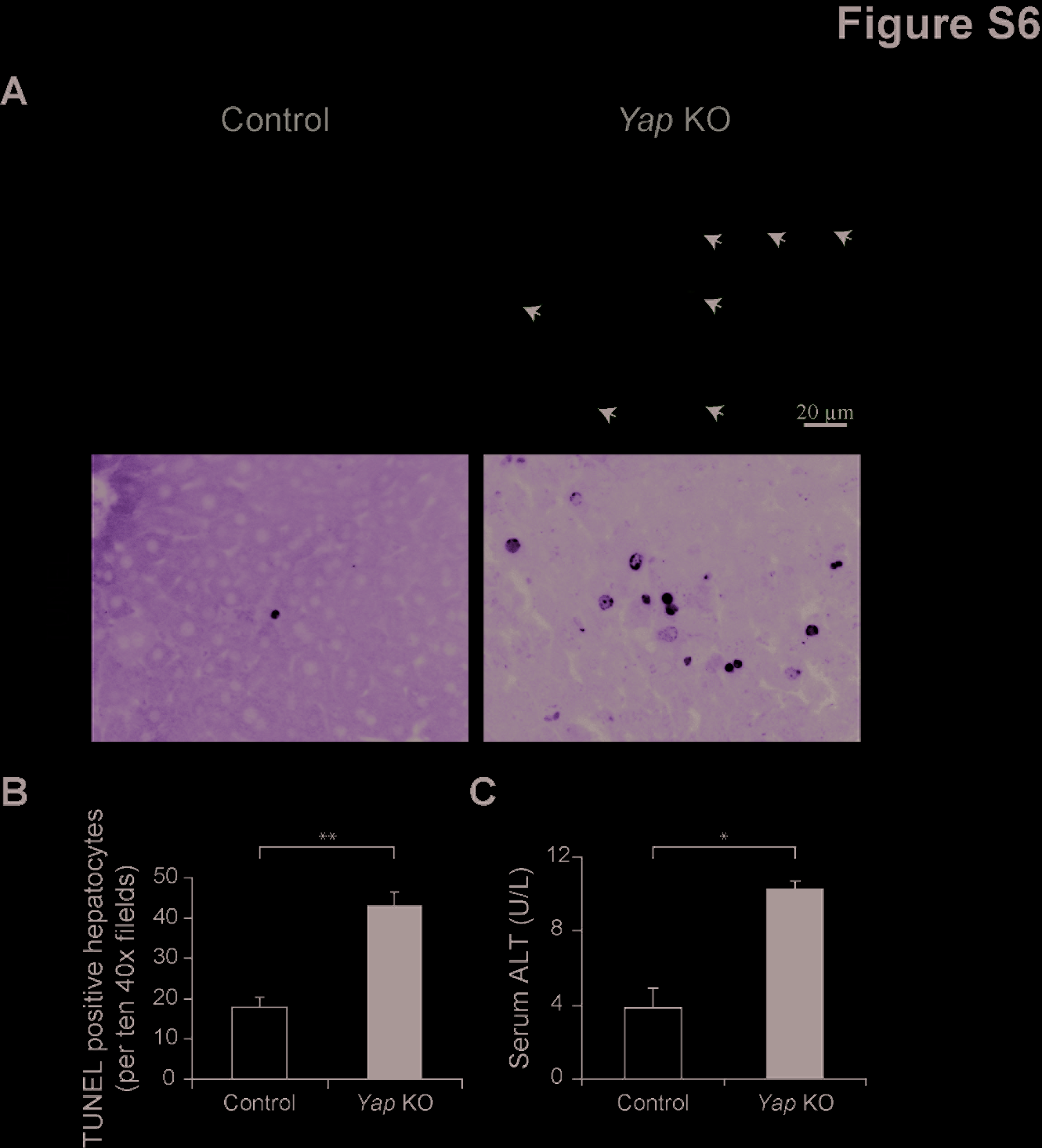

Supplement: Supplementary file 6 [file hep0056-1097-SD6.tif]
