# Supplementary material for: Yes-Associated Protein Regulates the Hepatic Response After Bile Duct Ligation
Source: Hepatology. 2012 Aug 8;56(3):1097–107. doi: 10.1002/hep.25769 (PMC3431197; doi:10.1002/hep.25769)
Supplement: Supplementary file 7 [file hep0056-1097-SD7.doc]

Primary Hepatocyte isolation and culture
 Hepatocytes were isolated by two-step collagenase perfusion of 8- to 12-week-old mice (37). The viability for freshly isolated hepatocytes was >90% by Trypan blue exclusion, and the purity was >95% by the unique morphology of hepatocytes at the time of proliferation assay started. Cells were plated at a density of 3000 cells per well of a collagen-coated 96-well plate and grown in D-MEM/F-12 medium supplemented with 1x ITS (Sigma), 30 µg/ml proline, 1mg/ml galactose, 7.5% NaHCO3, and 1x penicillin/streptomycin while incubated at 37°C with 5% CO2 as described (37). 30ng/ml epidermal growth factor (EGF) was added to the culture medium the next morning after isolation. The number of live cells was determined by the CellTiter 96® AQueous One Solution Cell Proliferation Assay (Promega, G3580). The experiments were performed in triplicate and repeated independently in three pairs of animals. 
Primary BEC isolation and culture
BECs were isolated according to the method of Vroman et al. (38). The viability of freshly isolated cholangiocytes was >90% by Trypan blue exclusion. The purity of the cholangiocytes was >90% by positive immunostaining to cytokeratin19 at the time of proliferation assay started. Isolated BECs were cultured and assayed as on collagen-coated plates in RPMI1640 medium supplemented with 1xITS, 3.6% FBS, 50ug/ml gentamicin and 1x penicillin/streptomycin at 37°C with 5% CO2. The cells at passage 3 were re-plated at a density of 5000 cells per well of a collagen-coated 96-well plate. 30ng/ml EGF was added to the culture medium the next morning after plating. The number of live cells was determined by the CellTiter 96® AQueous One Solution Cell Proliferation Assay (Promega, G3580). The experiments were performed in triplicate and repeated independently in three pairs of animals. 
Quantification of hepatocyte proliferation, BEC proliferation following BDL
To evaluate hepatocyte proliferation, the liver sections were stained with Ki67. Ten 10x objective fields per section for each mouse were randomly chosen and the Ki67 stained hepatocyte nuclei were counted for each 10x field. The number of Ki67 stained hepatocytes nuclei was calculated by summing the counts over ten fields. 
To evaluate BEC proliferation, the liver sections were co-stained with Ki67 and the BEC marker CK19. Ten 20x objective fields containing portal triads were evaluated for each sample and the ratio of Ki67+ BECs/total BECs was calculated. 
RNA isolation and Real-time PCR
Cellular RNA was extracted using RNeasy kit (Qiagen, #74104). RNA was reverse-transcribed with random primers using iScript cDNA synthesis kit (Bio-Rad, #170-8891). Real-time quantitative PCR (Q-PCR) was performed using the Quantitect SYBR Green PCR kit (Qiagen, #204143) on a StepOnePlus PCR system (Applied Biosystems). Q-PCR was done in triplicate, using histone H2A.Z as housekeeping control. Relative differences in the expression of the candidate genes in control and mutant livers were determined using the 2-ÄÄCT method. The mouse primer sequences used are available on request. 
